# Patients with Severe Radiographic Osteoarthritis Have a Better Prognosis in Physical Functioning after Hip and Knee Replacement: A Cohort-Study

**DOI:** 10.1371/journal.pone.0059500

**Published:** 2013-04-03

**Authors:** J. Christiaan Keurentjes, Marta Fiocco, Cynthia So-Osman, Ron Onstenk, Ankie W. M. M. Koopman-Van Gemert, Ruud G. Pöll, Herman M. Kroon, Thea P. M. Vliet Vlieland, Rob G. Nelissen

**Affiliations:** 1 Department of Orthopaedic Surgery, Leiden University Medical Center, Leiden, The Netherlands; 2 Department of Medical Statistics and BioInformatics, Leiden University Medical Center, Leiden, The Netherlands; 3 Department of Research and Development, Sanquin Blood Supply South West Region, Leiden, The Netherlands; 4 Department of Orthopaedic Surgery, Groene Hart Hospital, Gouda, The Netherlands; 5 Department of Anaesthesiology, Albert Schweitzer Hospital, Dordrecht, The Netherlands; 6 Department of Orthopaedic Surgery, Slotervaart Hospital, Amsterdam, The Netherlands; 7 Department of Radiology, Leiden University Medical Center, Leiden, The Netherlands; Tehran University of Medical Sciences, Islamic Republic of Iran

## Abstract

**Introduction:**

Although Total Hip and Knee Replacements (THR/TKR) improve Health-Related Quality of Life (HRQoL) at the group level, up to 30% of patients are dissatisfied after surgery due to unfulfilled expectations. We aimed to assess whether the pre-operative radiographic severity of osteoarthritis (OA) is related to the improvement in HRQoL after THR or TKR, both at the population and individual level.

**Methods:**

In this multi-center observational cohort study, HRQoL of OA patients requiring THR or TKR was measured 2 weeks before surgery and at 2–5 years follow-up, using the Short-Form 36 (SF36). Additionally, we measured patient satisfaction on a 11-point Numeric Rating Scale (NRSS). The radiographic severity of OA was classified according to Kellgren and Lawrence (KL) by an independent experienced musculoskeletal radiologist, blinded for the outcome. We compared the mean improvement and probability of a relevant improvement (defined as a patients change score≥Minimal Clinically Important Difference) between patients with mild OA (KL Grade 0–2) and severe OA (KL Grade 3+4), whilst adjusting for confounders.

**Results:**

Severe OA patients improved more and had a higher probability of a relevant improvement in physical functioning after both THR and TKR. For TKR patients with severe OA, larger improvements were found in General Health, Vitality and the Physical Component Summary Scale. The mean NRSS was also higher in severe OA TKR patients.

**Discussion:**

Patients with severe OA have a better prognosis after THR and TKR than patients with mild OA. These findings might help to prevent dissatisfaction after THR and TKR by means of patient selection or expectation management.

## Introduction

Total Hip Replacement (THR) and Total Knee Replacement (TKR) are effective surgical interventions, which alleviate pain and improve Health-Related Quality of Life (HRQoL) in patients with hip or knee joint degeneration at the population level. [1] Although on average patients improve markedly after THR or TKR, not all patients benefit from these surgeries. Persistent pain is reported in 9% of THR patients and 20% of TKR patients at long term follow-up. [2] Additionally, up to 30% of patients are dissatisfied after surgery, with higher reported dissatisfaction rates for TKR patients.[3]–[9] The relatively high dissatisfaction rate is especially worrying, as the therapeutic options are limited in dissatisfied patients after joint replacement. Moreover, given the projected increase in the annual number of THR and TKR performed in the United States, the absolute number of dissatisfied patients is expected to rise. [10].

Unattained expectations of surgery are thought to play an important role in dissatisfaction after joint replacement. [3], [4], [6], [11] In order to successfully manage patient expectations, accurate prediction of the probability of a meaningful improvement for each individual patient is of paramount importance. This probability can be assessed at the individual level using the Minimal Clinically Important Difference (MCID), which is defined as the minimal difference in scores of an outcome measure that is perceived by patients as beneficial or harmful. [12], [13] MCIDs in HRQoL, measured using the Short-Form 36, have been established for THR and TKR.[14]–[16].

Reports of the effect of the preoperative radiographic severity of osteoarthritis (OA) on the outcome of THR are conflicting: at the population level, Nilsdotter et al showed no effect at one year follow-up, while Meding et al found less postoperative pain at one year follow-up in patients with more preoperative joint space narrowing. [17], [18] At the individual level, patients with severe preoperative radiographic OA were more likely to improve in physical functioning. [19] We found no studies addressing the effect of the preoperative radiographic severity of osteoarthritis (OA) on the outcome of TKR.

From a clinical perspective, the preoperative radiographic severity of OA would be a helpful predictor of improvement in HRQoL, as it is both inexpensive and performed routinely for templating purposes. Moreover, the assessment of the severity of preoperative OA could be standardised, whereas this would be more difficult with subjective symptoms such as pain.

We questioned whether the radiographic severity of OA affects the improvement in HRQoL after THR and TKR, both at the population and individual level. Additionally, we questioned whether patient satisfaction with the surgical results differed between patients with mild or severe preoperative radiographical OA.

## Methods

We conducted a multi-center follow-up study at the departments of orthopaedic surgery of the Leiden University Medical Center, the Slotervaart hospital in Amsterdam, the Albert Schweitzer hospital in Dordrecht and the Groene Hart hospital in Gouda, the Netherlands, from August 2010 until August 2011. [20] The study was approved by the Medical Ethics Committee of the Leiden University Medical Center and the Medical Ethical Committees of all other participating centers; all patients gave written informed consent (CCMO-Nr: NL29018.058.09; MEC-Nr: P09.189). This study was registered in the Netherlands Trial Register (NTR2190). It concerned the clinical follow-up of a multi-center randomized controlled clinical trial, comparing different blood management modalities in THR and TKR surgery (Netherlands Trial Register: NTR303). In this trial, 2442 primary and revision hip or knee replacements in 2257 patients were included between 2004 and 2009.

All patients who participated in the randomized controlled trial and completed preoperative HRQoL questionnaires, who underwent primary THR of TKR for primary OA and who were alive at the time of inclusion for the present follow-up study were eligible for inclusion. In this study, patients are the subject of interest. Patients who participated more than once in the previous trial, were only allowed to participate once in the current study; the first joint replacement performed in the previous trial was chosen as the index surgery.

Records of the financial administration of all participating centers were checked in order to ascertain that all eligible patients were still alive before being approached. All eligible patients were first sent an invitation letter signed by their treating orthopaedic surgeon, an information brochure and a reply card. Patients who did not respond within 4 weeks after the first invitation were sent another invitation letter. The remaining patients, who did not respond to this second invitation, were contacted by telephone.

### Assessments

The assessments of the follow-up study consisted of patient-reported questionnaires, examination of patient records and preoperative radiographs.

#### Outcomes

HRQoL was measured preoperatively and in the present follow-up study using the SF36, which is translated and validated in the Dutch language. [21], [22] The 36 items cover eight domains (physical function, role physical, bodily pain, general health, vitality, social function, role emotional, and mental health), for which a sub-scale score is calculated (100 indicating no symptoms and 0 indicating extreme symptoms). Additionally, these scales are incorporated into two summary measures: a Physical Component Summary (PCS) and Mental Component Summary (MCS).

At the population level, the HRQoL outcome measure was the mean change score, i.e. the mean of each patients postoperative sub-scale score minus their pre-operative sub-scale score). At the individual level, the change scores were used to categorise patients in responders and non-responders, using previously published MCIDs.[14]–[16] Patients with a change score equal to or larger than the MCID of that particular sub-scale were categorised as a responder; patients whose change score was less than the CID of that particular sub-scale were categorised as non-responders.

Patient satisfaction with the surgical result was measured using an 11-point Numeric Rating Scale of Satisfaction (NRSS; 0 indicating completely dissatisfied, 10 indicating completely satisfied). At the population level, the satisfaction outcome measure was the mean NRSS score. The proportion of patients who achieved a satisfactory outcome (defined as a NRSS>8, according to Brokelman et al [5]) was the satisfaction outcome measure at the individual level.

#### Exposure

Pre-operative radiographs of the hips (anterior–posterior) and knees (posterior–anterior) were collected from the participating patients' medical records and radiology department. These radiographs were routinely made in each participating center for pre-operative templating purposes. All radiographs were assessed by an experienced musculoskeletal radiologist (HMK), who was blinded for patient characteristics and HRQoL assessments. The method of scoring OA followed that described by Kellgren and Lawrence (KL) (0 indicating no OA, 1 doubtful OA, 2 minimal OA, 3 moderate OA and 4 indicating severe OA). [23] All radiographs were scored twice: both readings were used to establish intra-reader reliability (Intra-Class Correlation hip radiographs: 0.85 (95%CI: 0.82–0.88); Intra-Class Correlation knee radiographs: 0.87 (95%CI: 0.83–0.89)). The second reading was used for further statistical analyses.

As KL grade 0 to 2 and grade 3 and 4 are deemed similar from a clinical perspective, we grouped the severity of pre-operative OA in 2 categories: mild radiographic OA (KL grade 0, 1 or 2) and severe radiographic OA (KL grade 3 or 4).

#### Potential confounders

Socio-demographic characteristics collected at baseline in the trial included: age at joint replacement and gender. Additionally, the following socio-demographic variables were collected in the questionnaire of the follow-up study: length and weight, in order to calculate the Body Mass Index (BMI) (<25, 25–30, 30–35, >35) and patient reported Charnley classification of co-morbidity (Class A: patients in which the index operated hip or knee are affected only; Class B: patients in which the other hip or knee is affected as well; Class C: patients with a hip or knee replacement and other affected joints and/or a medical condition which affects the patients' ability to ambulate). [24], [25].

### Statistical Analysis

We performed descriptive analyses of patients baseline characteristics. In order to investigate the possible extent of self-selection bias, we compared the age at THR or TKR and gender of participants to non-participants.

Patients with missing pre-operative SF36 questionnaires, missing SF36 questionnaires at follow-up or missing pre-operative radiographs were excluded from analyses, as we could not exclude a Missing Not At Random (MNAR) mechanism. Missing values of the Charnley Co-morbidity Classification and BMI were deemed Missing At Random and imputed using Multiple Imputations (MI), in order to improve efficiency of the regression analyses and avert biased regression coefficients. We performed MI (m = 10) using an Expectation-Maximization algorithm, [26] which is implemented in the Amelia 2 package for R. [27], [28].

We performed regression analyses in each imputed dataset in order to compare the mean improvement in HRQoL and the probability of achieving a MCID in HRQoL after THR and TKR, between patients with KL grade 0, 1 or 2 and grade 3 or 4. As MCIDs in HRQoL differ between THR patients and TKR patients, we performed all analyses separately for THR and TKR. Possible confounders are age, gender, BMI and poly-articular OA in both THR and TKR patients. We used the Charnley classification as a proxy for poly-articular OA. As the length of follow-up varied considerably, we first stratified our data in quartiles of follow-up length for each imputed dataset. Within each stratum of follow-up length, we performed a multivariate mixed effect linear regression analysis, with the mean improvement in HRQoL and the mean NRSS as the dependent variable, the KL grade and confounders as independent variables and center as a random effect. Stratum-specific mean differences in HRQoL between the KL grades were pooled using inverse variance weighting in order to produce an overall estimate of the mean difference in HRQoL for each imputed data-set. Finally, the *m = 10* estimates of the mean differences in HRQoL were combined into one estimate, according to Rubin. [29].

Within each stratum of follow-up length, we also performed a multivariate mixed effect logistic regression analysis, with the probability of attaining a MCID in HRQoL and a satisfactory NRSS as the dependent variable, the KL grade and confounders as independent variables and center as a random effect. Stratum-specific odds ratios of attaining a MCID in HRQoL between the KL grades were pooled using inverse variance weighting in order to produce an overall estimate of the odds ratio of attaining a MCID in HRQoL for each imputed data-set. Finally, the *m = 10* estimates of the mean differences in HRQoL were combined into one estimate, according to Rubin. [29].

All analyses were performed using R, version 2.14.0. [30].

## Results

**Figure 1:**
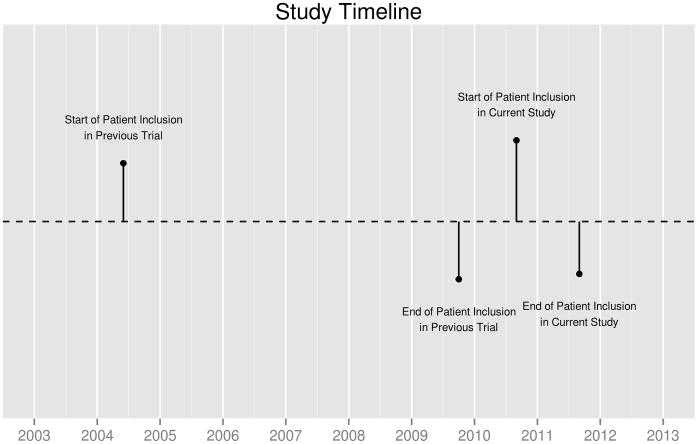
Study Timeline.

**Figure 2:**
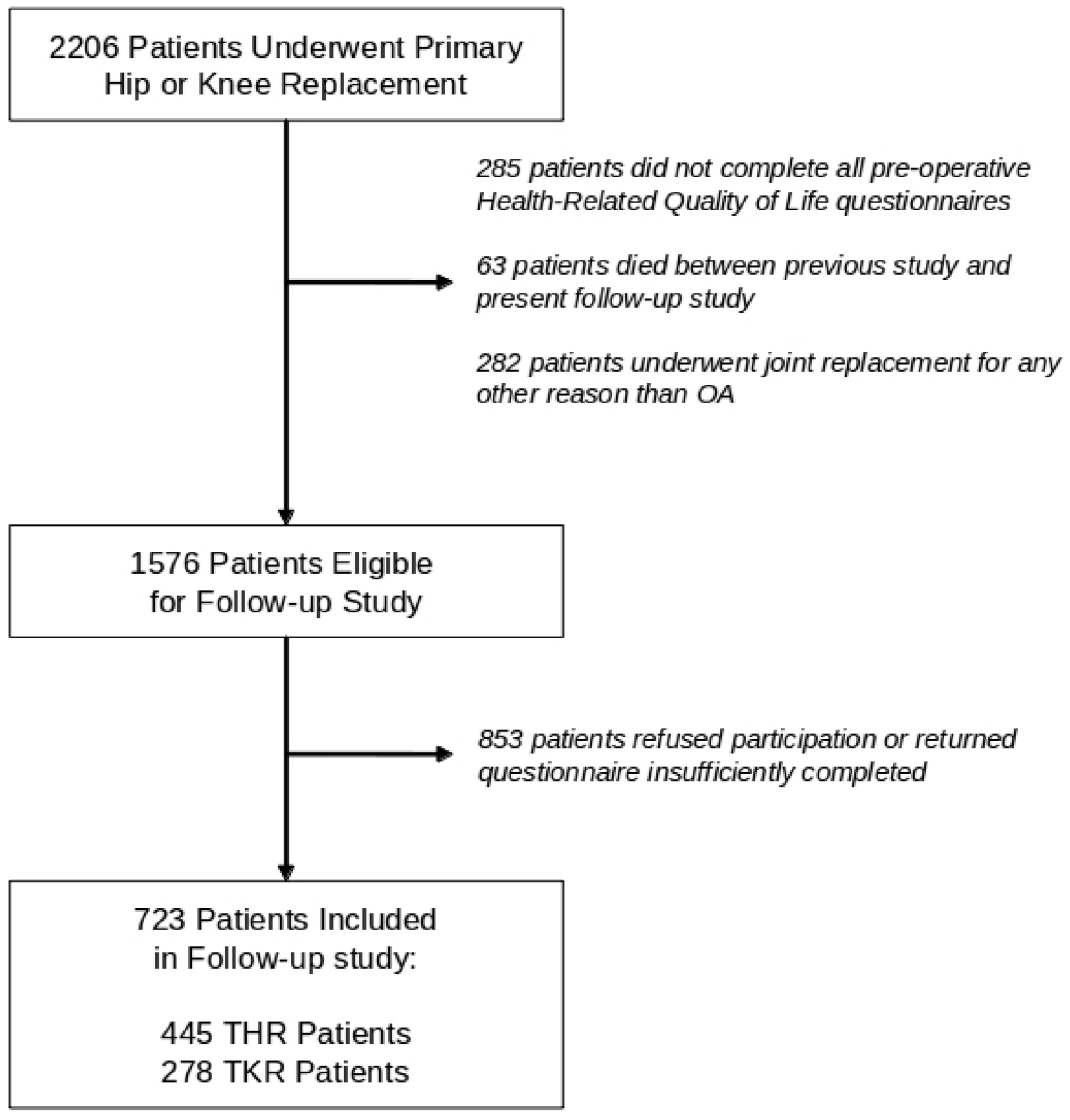
Patient Inclusion Flow-chart.

At 2 to 5 years after joint replacement, 723 patients agreed to participate and returned the questionnaires sufficiently completed (participation rate: 46%, figure 1 and 2). Non-participating THR patients were on average 4.32 years older than participants (95%CI: 2.93–5.70 years); Non-participating TKR patients were on average 2.68 years older than participants (95%CI: 1.28–4.09 years). The proportion of males was similar in participants and non-responders. An overview of the patient characteristics is provided in table 1.

**Table 1:** Patient Characteristics.

Primary THR	Kellgren Grade 0–2	Kellgren Grade 3+4	All Patients
Age at Joint Replacement	65.1 (7.8)	67.4 (8.7)	66.6 (8.5)
Males	23.9%	44.3%	37.5%
Follow-up (years)	2.83 (1.0)	2.79 (0.9)	2.8 (0.93)
Charnley:			
Class A:	25.8%	24.0%	24.6%
Class B:	14.6%	13.7%	14.0%
Class C:	59.6%	62.3%	61.4%
Body Mass Index:			
<25:	29.2%	34.1%	32.5%
25–30:	41.6%	46.4%	44.8%
30–35:	23.6%	16.2%	18.7%
>35:	5.60%	3.40%	4.10%
**Primary TKR**	**Kellgren Grade 0–2**	**Kellgren Grade 3+4**	**All Patients**
Age at JointReplacement	65.1 (10.3)	69.5 (8.6)	69.1 (8.9)
Males	31.8%	30.9%	31.0%
Follow-up (years)	3.1 (1.1)	2.79 (0.9)	2.82 (0.93)
Charnley:			
Class A:	4.50%	19.0%	17.5%
Class B:	4.50%	11.4%	10.7%
Class C:	90.9%	69.6%	71.8%
Body MassIndex:			
<25:	33.3%	14.7%	16.3%
25–30:	27.8%	46.7%	45.0%
30–35:	33.3%	21.2%	22.3%
>35:	5.60%	17.4%	16.3%

All values are mean (SD), unless stated otherwise.

In 13 THR patients and 7 TKR patients, the Charnley classification was missing; in 9 THR patients and 11 TKR patients, the BMI was missing. These missing values were imputed using multiple imputation.

The mean improvement in HRQoL and mean NRSS per KL grade is shown in table 2 for THR patients and table 3 for TKR patients. In THR, patients with severe radiographic OA had a larger improvement in Physical Functioning than patients with mild radiographic OA. The improvement in other domains of HRQoL and the mean NRSS was similar for THR patients of all severities of radiographic OA. In TKR, patients with severe radiographic OA had a larger improvement in Physical functioning than patients with mild radiographic OA. Additionally, patients with severe radiographic OA had a larger improvement in General Health, a larger improvement in the Physical Component Summary Scale and a higher NRSS than patients with mild radiographic OA.

**Table 2:** Improvement in Health-Related Quality of Life and Satisfaction after Hip Replacement: A Comparison Between Patients with Mild to Moderate and Severe Radiographical Pre-Operative Osteoarthritis.

SF36 Sub-Scale	Kellgren Grade 0–2:Mean Improvement (95%CI)	Kellgren Grade 3+4:Mean Improvement (95%CI)	Kellgren Grade 0–2 VS Kellgren Grade 3+4:Mean Adjusted Difference (95%CI)	P-value
Physical Functioning	19.2 (14.2–24.1)	26.2 (22.4–30.0)	8.93 (2.14–15.7)	*0.01*
Role-Physical	36.3 (26.7–45.9)	42.2 (35.4–48.9)	6.39 (−5.89–18.7)	0.31
Bodily Pain	35.9 (30.4–41.3)	36.5 (32.8–40.2)	0.88 (−6.08–7.84)	0.80
General Health	0.60 (−3.50–4.60)	−1.50 (−4.50–1.50)	−0.66 (−5.66–4.34)	0.79
Vitality	9.30 (5.00–13.5)	3.70 (0.80–6.70)	−3.53 (−9.03–1.97)	0.21
Social Functioning	19.4 (13.6–25.2)	14.6 (10.7–18.4)	−4.11 (−11.2–2.97)	0.25
Role Emotional	6.90 (−1.10–14.9)	11.3 (4.70–17.8)	3.11 (−8.22–14.4)	0.59
Mental Health	7.20 (4.00–10.5)	4.60 (2.10–7.10)	−1.80 (−6.13–2.50)	0.41
PCS	10.7 (8.70–12.6)	11.2 (9.90–12.6)	1.94 (−0.57–4.44)	0.13
MCS	1.50 (−0.40–3.40)	−0.50 (−1.80–0.90)	−2.03 (−4.46–0.39)	0.10
NRS Satisfaction	8.5 (8.0–8.9)	8.9 (8.6–9.2)	0.3 (−0.2–0.9)	0.19

Positive values indicate a higher mean improvement in HRQoL after THR in patients with Kellgren Grade 3+4, compared to Grade 0–2.

The mean differences between radiographic severity are adjusted for age, sex, Charnley Comorbidity Classification and BMI and stratified for quartiles of follow-up.

**Table 3:** Improvement in Health-Related Quality of Life and Satisfaction after Knee Replacement: A Comparison Between Patients with Mild to Moderate and Severe Radiographical Pre-Operative Osteoarthritis.

SF36 Sub-Scale	Kellgren Grade 0–2:Mean Improvement (95%CI)	Kellgren Grade 3+4:Mean Improvement (95%CI)	Kellgren Grade 0–2 VS Kellgren Grade 3+4:Mean Adjusted Difference (95%CI)	P-value
Physical Functioning	−2.10 (−10.5–6.30)	15.1 (11.7–18.5)	19.1 (8.48–29.7)	*<0.001*
Role-Physical	9.10 (−11.9–30.1)	20.6 (−13.5–27.7)	17.4 (−6.32–41.1)	0.15
Bodily Pain	14.5 (3.50–25.5)	25.2 (21.5–29.0)	9.02 (−3.43–21.5)	0.15
General Health	−9.10 (−16.9– −1.30)	−1.50 (−3.80–0.80)	9.23 (1.31–17.2)	*0.02*
Vitality	−5.40 (−13.0–2.30)	1.20 (−1.40–3.80)	8.44 (−0.28–17.2)	0.06
Social Functioning	2.80 (−8.00–13.6)	8.90 (5.40–12.4)	7.44 (−4.18–19.1)	0.21
Role Emotional	4.50 (−17.5–26.6)	5.80 (−0.60–12.1)	8.87 (−11.8–29.6)	0.40
Mental Health	3.40 (−4.00–10.8)	3.00 (0.80–5.10)	0.29 (−6.93–7.50)	0.94
PCS	1.50 (−2.90–6.00)	6.40 (5.10–7.70)	5.64 (1.26–10.0)	*0.01*
MCS	0.10 (−4.30–4.40)	−0.30 (−1.60–1.00)	−0.18 (−4.45–4.10)	0.94
NRS Satisfaction	7.4 (6.1–8.6)	8.2 (7.9–8.6)	1.2 (0.1–2.4)	*0.04*

Positive values indicate a higher mean improvement in HRQoL after THR in patients with Kellgren Grade 3+4, compared to Grade 0–2. The mean differences between radiographic severity are adjusted for age, sex, Charnley Comorbidity Classification and BMI and stratified for quartiles of follow-up.

The crude probabilities of achieving a MCID in each dimension of HRQoL are presented in table 4 for THR patients and table 5 for TKR patients. In THR, the probability of achieving a relevant improvement in Physical Functioning was higher in patients with severe radiographic OA than in patients with mild radiographic OA. The probability of achieving a satisfactory outcome was also higher in patients with severe radiographic OA than in patients with mild radiographic OA. The probability of achieving a relevant improvement in other domains of HRQoL was similar for THR patients of all severities of radiographic OA. In TKR, the probability of achieving a relevant improvement in Physical Functioning was higher in patients with severe radiographic OA than in patients with mild radiographic OA. Additionally, the probability of achieving a relevant improvement in General Health and the probability of achieving a satisfactory outcome was also higher in patients with severe radiographic OA than in patients with mild radiographic OA.

**Table 4:** Improvement in Health-Related Quality of Life and Satisfaction after Hip Replacement: A Comparison Between Patients with Mild to Moderate and Severe Radiographical Pre-Operative Osteoarthritis.

SF36 Sub-Scale	Kellgren Grade 0–2:Probability of Achieving a MCID (95%CI)	Kellgren Grade 3+4:Probability of Achieving a MCID (95%CI)	Kellgren Grade 0–2 VS Kellgren Grade 3+4:Adjusted Odds Ratio (95%CI)	P-value
Physical Functioning	64/92∶69.6% (59.5–78.0)	146/185∶78.9% (72.5–84.2)	1.87 (0.97–3.60)	0.06
Role-Physical	55/92∶59.8% (49.6–69.2)	124/185∶67.0% (60.0–73.4)	1.50 (0.82–2.72)	0.19
Bodily Pain	71/92∶77.2% (67.6–84.6)	141/185∶76.2% (69.6–81.8)	1.03 (0.52–2.05)	0.93
General Health	62/92∶67.4% (57.3–76.1)	117/185∶63.2% (56.1–69.9)	0.91 (0.47–1.77)	0.78
Vitality	34/92∶37.0% (27.8–47.2)	54/185∶29.2% (23.1–36.1)	0.84 (0.46–1.55)	0.58
Social Functioning	42/92∶45.7% (35.9–55.8)	80/185∶43.2% (36.3–50.4)	0.87 (0.49–1.55)	0.64
Role Emotional	21/92∶22.8% (15.4–32.4)	51/185∶27.6% (21.6–34.4)	1.01 (0.51–2.01)	0.98
Mental Health	17/92∶18.5% (11.9–27.6)	40/185∶21.6% (16.3–28.1)	1.26 (0.62–2.58)	0.53
NRS Satisfaction >8	53/92∶57.6% (47.4–67.2)	136/185∶73.5% (66.7–79.3)	1.95 (1.06–3.59)	*0.03*

Odds Ratios >1 indicate a higher probability of achieving a Minimal Clinically Important Difference in HRQoL after THR in patients with Kellgren Grade 3+4, compared to Grade 0–2.

The odds ratios adjusted for age, sex, Charnley Comorbidity Classification and BMI and stratified for quartiles of follow-up.

**Table 5:** Improvement in Health-Related Quality of Life and Satisfaction after Knee Replacement: A Comparison Between Patients with Mild to Moderate and Severe Radiographical Pre-Operative Osteoarthritis.

SF36 Sub-Scale	Kellgren Grade 0–2:Probability of Achieving a MCID (95%CI)	Kellgren Grade 3+4:Probability of Achieving a MCID (95%CI)	Kellgren Grade 0–2 VS Kellgren Grade 3+4:Adjusted Odds Ratio (95%CI)	P-value
Physical Functioning	5/22∶22.7% (10.1–43.4)	105/191∶55.0% (47.9–61.9)	5.44 (1.45–20.3)	*0.01*
Role-Physical	9/22∶40.9% (23.3–61.3)	88/191∶46.1% (39.2–53.2)	1.46 (0.49–4.32)	0.50
Bodily Pain	15/22∶68.2% (47.3–83.6)	136/191∶71.2% (64.4–77.2)	1.15 (0.32–4.16)	0.83
General Health	9/22∶40.9% (23.3–61.3)	122/191∶63.9% (56.9–70.4)	3.56 (1.23–10.4)	*0.02*
Vitality	8/22∶36.4% (19.7–57)	86/191∶45.0% (38.1–52.1)	1.09 (0.35–3.44)	0.88
Social Functioning	7/22∶31.8% (16.4–52.7)	98/191∶51.3% (44.3–58.3)	2.84 (0.87–9.32)	0.08
Role Emotional	6/22∶27.3% (13.2–48.2)	41/191∶21.5% (16.2–27.8)	0.85 (0.26–3.02)	0.85
Mental Health	8/22∶36.4% (19.7–57)	79/191∶41.4% (34.6–48.4)	2.79 (0.70–11.2)	0.15
NRS Satisfaction >8	9/22∶40.9% (23.3–61.3)	116/191∶60.7% (53.7–67.4)	2.25 (0.78–6.52)	0.14

Odds Ratios >1 indicate a higher probability of achieving a Minimal Clinically Important Difference in HRQoL after THR in patients with Kellgren Grade 3+4, compared to Grade 0–2.

The odds ratios adjusted for age, sex, Charnley Comorbidity Classification and BMI and stratified for quartiles of follow-up.

## Discussion

At the population level, patients with severe radiographic OA improve more in Physical Functioning than patients with mild radiographic OA, both for THR and TKR. At the individual level, THR and TKR patients with severe radiographic OA have a larger probability of a relevant improvement in Physical Functioning than patients with mild radiographic OA. The effects of the preoperative severity of radiographic OA on Physical Functioning are more pronounced in TKR patients than in THR patients. Other domains of HRQoL do not appear to be influenced by the preoperative severity of OA, except General Health and the Physical Component Summary Scale in TKR patients. Additionally, patient satisfaction appears to be better in patients with more severe preoperative radiographic OA.

Limitations of the study include the participation rate and range of follow-up period after joint replacement. Although participation rates of 100% are feasible in small-scaled studies with hard endpoints, [31], [32] participation rates in epidemiological studies have been steadily declining in the last 30 years. [33] Even sharper declines have been reported in the past few years. [34] Unfortunately, the participation rate of this study follows this general trend, resulting in a participation rate of 46%. Therefore, we cannot exclude the presence of self-selection bias. In order to limit the extent of this bias, we have sent multiple reminders and have called all patients who did not answer our reminders and who did not return the questionnaire. As incentives, we have included an appealing information brochure in which the primary goals of the follow-up study were explained and a study pen as a small gift. Additionally, patients were urged to participate by their treating physician. However, the participation rate alone does not determine the extent of bias present in any particular study. [34] The difference between participants and non-participants is far more important. [35] As the found differences in demographics were of little clinical relevance, it is unlikely that the study results will be severely biased. Finally, the patient demographics of our study population were similar to those of large-scaled national joint registry studies, regarding age, gender, Charnley classification and BMI. [36], [37].

The follow-up period after joint replacement varies between 2 and 5 years. Although a residual effect of follow-up length cannot be excluded, we do not think this is very plausible, as recent evidence suggests that the improvement in HRQoL is sustained up to 5 years after joint replacement surgery. [38], [39].

Although joint replacements are highly effective in improving HRQoL at the group level, [1] this is not the case for each individual patient, judging from the relatively high dissatisfaction rates. [40], [41] Studying HRQoL at the individual level, using the probability of achieving a clinically important difference as an outcome measure, enables a better prediction of a successful outcome. Moreover, it could provide a helpful way to fine-tune the indication for joint replacement, for which there are no clear cut-off points currently available. [42].

Regardless of age, gender, co-morbidity and BMI, we have shown that joint replacement patients with severe preoperative OA have a better prognosis in improvement in Physical Functioning and patient satisfaction with the surgical results. These effects are more pronounced in TKR patients than in THR patients, which might be explained in part by biomechanical factors. The hip joint is a relatively simple ball and socket joint, which is adequately mimicked by a THR. The biomechanical aspects of the knee joint are more difficult to imitate, as the knee is a pivotal hinge joint with 6 degrees of freedom. These degrees of freedom are generally not restored after TKR, which is substantiated in kinematic and kinetic studies. [43] This additional disadvantage of TKR patients who underwent joint replacement for mild radiographic OA is reflected in a smaller increase in Physical Functioning than THR patients who underwent joint replacement for mild radiographic OA. Additionally, the odds of achieving a MCID in Physical Functioning is smaller and the difference in satisfaction is larger.

Clinically, these are promising findings, as dissatisfaction rates are higher in TKR patients than in THR patients. [4], [6] Patient satisfaction is thought to be closely related to unfulfilled expectations. Although patient expectations of THR and TKR are similar, recent evidence suggests that THR meets important patient expectations better than TKR. [6], [11], [44] Our findings could lead to a more fitting expectation management regarding the expected improvement in Physical Functioning, using a single predictor. This improvement in expectation management might lead to higher satisfaction rates.

Plain radiographs have a number of appealing aspects. In the first place, they are inexpensive and easily available, as they are currently a part of the clinical work-up to joint replacement. Secondly, due to the non-invasive character of the test, radiographs are a patient-friendly modality. Finally, they offer a more objective approach to joint complaints. These aspects would make it easy to implement the KL grade in clinical practice, in order to predict HRQoL and satisfaction after joint replacement.
